# Uncovering the Missing Pieces: Predictors of Nonresponse in a Mobile Experience Sampling Study on Media Effects Among Youth

**DOI:** 10.1177/08944393241235182

**Published:** 2024-02-23

**Authors:** Anne Reinhardt, Sophie Mayen, Claudia Wilhelm

**Affiliations:** 1 27258 University of Vienna, Austria

**Keywords:** mobile experience sampling, non-response, missing data handling, adolescents, displacement effects, media use

## Abstract

Mobile Experience Sampling (MES) is a promising tool for understanding youth digital media use and its effects. Unfortunately, the method suffers from high levels of missing data. Depending on whether the data is randomly or non-randomly missing, it can have severe effects on the validity of findings. For this reason, we investigated predictors of non-response in an MES study on displacement effects of digital media use on adolescents’ well-being and academic performance (*N* = 347). Multilevel binary logistic regression identified significant influencing factors of response odds, such as afternoon beeps and being outside. Importantly, adolescents with poorer school grades were more likely to miss beeps. Because this missingness was related to the outcome variable, modern missing data methods such as multiple imputation should be applied before analyzing the data. Understanding the reasons for non-response can be seen as the first step to preventing, minimizing, and handling missing data in MES studies, ultimately ensuring that the collected data is fully utilized to draw accurate conclusions.

The use of digital media by adolescents has become ubiquitous, transcending temporal and spatial barriers. With almost every western teenager owning a smartphone, they are constantly within reach of various online activities, including social media, multimedia consumption, and gaming (for Germany, see Feierabend et al., 2022). How digital media use impacts adolescents’ lives is a subject of intense debate. The displacement hypothesis postulates that digital media use could have negative consequences on essential social outcomes, such as academic performance or well-being (for an overview of the ongoing discussion, see, e.g., Valkenburg, 2022). However, approaching this relationship is complicated by the fact that accurately measuring youth digital media use presents a significant challenge in our increasingly media-saturated world.

Conventional cross-sectional surveys that rely on retrospective Likert scales to measure media use can suffer from memory gaps and fail to capture short-term usage periods (Naab et al., 2019). The Mobile Experience Sampling (MES) method offers a compelling alternative (for studies on media use and effects, see, e.g., Otto et al., 2020; Schnauber-Stockmann & Karnowski, 2020; Siebers et al., 2021). In MES studies, the data is collected on a mobile device (e.g., smartphone) over an extended observation period through self-reports (van Berkel & Kostakos, 2021). Unlike traditional daily diary studies, the participants receive multiple daily prompts (so-called “beeps”) to fill in the questionnaire, proactively triggered by the researchers (van Berkel et al., 2017). For each prompt, participants indicate what they were doing directly or shortly before and, thus, do not have to rely on their long-term memory (in situ measurement; Karnowski, 2013).

While the MES method offers numerous advantages over traditional survey data, it is not without flaws. One significant drawback is the relatively low completion rates, with studies reporting figures ranging between 70%–85% (Stone et al., 2023; Sun et al., 2021). Compliance in MES studies, defined as “the percentage of beeps responded to of the total number requested” (Stone et al., 2023, p. 1.13), poses a significant challenge, particularly given that the survey period usually spans several days or weeks, with multiple prompts per day. If the missingness is related to the outcome variable of interest—therefore missing at a nonrandom manner—it can lead to biased and skewed findings (Pedersen et al., 2017). Hence, it is important to identify predictors of missingness in order to decide how to evaluate and handle missing data in MES studies.

Unfortunately, research on determinants of non-response in MES studies is limited, whereby the following two trends can be identified: (1) While most studies focus on drug use (e.g., Markowski et al., 2021; Messiah et al., 2010), physical activity (e.g., McLean et al., 2017), or emotions (e.g., Courvoisier et al., 2012; Rintala et al., 2020; Sun et al., 2021), there is a significant gap in understanding non-response in the context of media use and effects research. This gap is especially critical for factors related to variables in the context of the displacement hypothesis, such as media use and its outcomes (e.g., well-being, academic performance). (2) Most of the literature focuses on adult samples, ignoring the unique needs and characteristics of adolescents. For instance, while adults dispose freely of their media devices, adolescents’ media use is often regulated; thus, contextual factors might be more important among adolescents compared to adults.

The article delves into the factors influencing whether adolescents respond to prompts in an MES study. In detail, we analyze both predictors on the beep level (i.e., factors observed at each prompt), such as the setting and context of the study, and person-level determinants (i.e., factors observed once at the baseline survey), like academic performance and well-being. Understanding the reasons for non-response can be seen as the first step to preventing, minimizing, and handling missing data in MES studies, ultimately ensuring that the collected data is fully utilized to draw accurate conclusions.

## Understanding Missing Data in MES Studies

Rubin (1976) distinguishes three categories of missingness. First, missing completely at random (MCAR) implies that the missing data is unrelated to the measured variables, which leads to less power but unbiased findings. However, MCAR is often an “ideal but unreasonable assumption” (Kang, 2013, p. 405). Second, missing at random (MAR) indicates that the missing data can be explained by other observed measures but is unrelated to the specific outcomes expected to be obtained. Third, missing not at random (MNAR) refers to the missingness that is systematically related to unobserved data.

In MES studies, missing data occurs more frequently at the beep level than the between-person level (Silvia et al., 2013). The reasons behind missing data are multifaceted and can be related to either technological or situational factors (Stone et al., 2023). Technological factors may include problems in data transmission or collection, such as poor Internet connection or switched-off smartphones, which results in MCAR/MAR data. On the other hand, situational factors may arise when participants forget to respond due to distraction or are unable to respond because of the situation they are in (e.g., when in class). Such factors typically lead to MAR data, where missing data is attributed to observed variables not related to the outcome variable. Nonetheless, in certain cases, missing MES data can be MNAR, implying that the willingness to answer a prompt is associated with the outcome variable itself. For example, when measuring well-being, participants with depressive symptoms might have a higher probability of non-response due to their mental state, which would bias the findings (e.g., Vachon et al., 2019).

In terms of data analysis, the type of missingness determines the missing data handling method of choice: While for data MCAR/MAR researchers might decide to conduct a complete case analysis, list-wise deletion of missing cases can lead to biased and skewed findings when the data is missing in a nonrandom manner (Pedersen et al., 2017). In such cases, modeling missing data (e.g., through multiple imputation) is the only appropriate way to respond. However, multiple imputation requires that the causes or correlates of missingness are known (Kang, 2013). Hence, identifying predictors of missingness is of great importance (Courvoisier et al., 2012; Grund et al., 2018; Silvia et al., 2013). Subsequently, we present a summary of the current literature on predictors of missing data in MES research and derive the hypotheses and research questions of this study.

## Predictors of Non-Response in MES Studies

Understanding the reasons behind non-response is critical for improving the reliability and validity of MES findings. As outlined above, predictors of non-response can be allocated at the beep level, considered as within-subjects factors, and on the person-level, considered as between-subjects factors. We first discuss effects of beep level predictors, followed by predictors of non-response on the person-level.

### Beep Level

#### Study Settings

The study day seems to play an important role in predicting response to a MES prompt. Research has shown that missing data tends to increase over the course of a study, which is attributed to a decreased motivation and fatigue symptoms (e.g., McLean et al., 2017; Rintala et al., 2020; Silvia et al., 2013; Sokolovsky et al., 2014; Sun et al., 2021). However, other studies indicate that it is not solely the study length that predicts non-response but more the specific day itself. For instance, Broda (2017) demonstrated that high school students were more likely to ignore a beep when it occurred at the weekend (for a similar finding among adults, see Markowski et al., 2021). However, another study found university students’ odds of non-response were higher in the middle of the week compared to the beginning or end of the week (Martinez et al., 2021). No effects were found by Sun et al. (2021) and Courvoisier et al. (2012). Hence, the following research question is put forward:

RQ1 How does the study day affect the odds of non-response?

Mixed findings were also found for the impact of the scheduled time of the prompt. Studies among adults have suggested that the likelihood of non-response increased when the prompt was received in the morning (Courvoisier et al., 2012; Messiah et al., 2010; Sun et al., 2021) or in the evening (Rintala et al., 2020). Interestingly, a study among school students found that beeps occurring after school had higher odds of being missed than those in the morning (Broda, 2017). To clarify whether this finding is unique to the student population or influenced by other factors, we ask:

RQ2 How does the time of the prompt affect the odds of non-response?

#### Time-Lagged Factors

While factors such as study day and scheduled prompt time have already been examined, little is known about what individuals were doing when missing a beep. To shed light on this question, researchers have employed lagged predictor models to explore how prior experiences influence the likelihood of non-response at the following beep (Sun et al., 2021).

Despite their potential, only a handful of studies have used these models, revealing that both psychological states and contextual factors play a role in response behavior (Rintala et al., 2020; Silvia et al., 2013; Sokolovsky et al., 2014). In a MES study among 450 students, Silvia et al. (2013) examined the influence of time-lagged emotional states, level of fatigue, and context (being alone vs. around others), showing that only the strength of enthusiasm significantly predicted the response likelihood. Based on their findings, the authors conclude that within-day experiences are secondary when explaining compliance in MES studies. In contrast, based on time-lagged data from more than 1000 adults, Rintala et al. (2020) found that participants who felt disturbed at the prior beep or were outside their homes had higher odds of non-response at the following prompt. To our knowledge, there is only one study using time-lagged analysis in a youth sample: In a longitudinal survey on smoking escalation, Sokolovsky et al. (2014) found positive affect and being outside their home at the prior beep increased adolescents’ chance of non-compliance at the next signal.

As can be seen, research taking a time-lagged perspective is scarce and leaves out essential factors related to media use and effects. Beside contextual factors, we will investigate the influence of affective well-being and flow experience (i.e., being completely absorbed in an activity) on the response odds at the subsequent beep, since both play a role in the context of media displacement effects among adolescents (e.g., Dienlin et al., 2017; Hall et al., 2019; Smith et al., 2017). Based on the literature, the following hypothesis and research questions are put forward:

H1 Adolescents (a) outside their home and (b) around others at the prior beep have increased odds of non-response at the next beep.

RQ3 How does time-lagged affective well-being influence the odds of non-response?

RQ4 How does time-lagged flow experience influence the odds of non-response?

#### Person-Level

The odds of non-response are also influenced by individual characteristics such as sociodemographic and socioeconomic variables, personality traits, and medical conditions (Martinez et al., 2021; McLean et al., 2017; Messiah et al., 2010; Rintala et al., 2020; Silvia et al., 2013). Amongst others, non-response can be associated with being male, being a drug user, high levels of physical activity, being younger, and being a non-native speaker.

Studies investigating the effects of person-level variables on response odds among adolescents are scarce. Broda (2017) observed no effects of gender, race/ethnicity, and school assignment, while higher levels of boredom increased the likelihood of non-response. Sokolovsky et al. (2014) found that higher mean negative affect, smoking rate, alcohol use, and male gender predicted lower compliance with MES prompts.

Based on the current literature, it remains unclear how person-level factors related to adolescents’ socioeconomic and cultural background, media use, well-being, and academic performance influence the odds of non-response in MES studies in the context of media use and effects research. We therefore ask:

RQ5 How do (a) sociodemographic variables, (b) socioeconomic factors, (c) media use, (d) academic performance, and (e) overall well-being influence the odds for non-response?

## Method

### Sampling Procedures

Before data collection, the project was pre-registered on OSF (https://osf.io/hej54/?view_only=204d84a6314f414495f403eaf968fddd)^1^ and approved by the ethics committee of the University of Vienna (#00776). Study participants were recruited from nine schools in two urban cities in Austria, representing different types of schools (middle school, secondary school). Eligible students had to be at least 12 years old. The data collection took place September to November 2022.

### Design and Procedure

The study consisted of two parts, that is, an online baseline survey and a one-week MES. Before data collection, the students received study information material and were briefed about the procedures. Then, we collected the informed consent. For students under 14 years, informed consent was provided by their parents.

The beeps were sent via text message on participants’ smartphones, providing a link to the questionnaire. In the week of data collection, the 15-min baseline survey took place on Monday (5 pm) and measured all relevant between-person variables. Afterward, participants received three prompts each on two working days (Tue, Wed) and one weekend day (Sat). The MES survey (∼3 min) asked about their media use and experiences (e.g., well-being, flow) during the last hour. The beeps were scheduled at 2 pm, 4.30 pm, and 7 pm on working days. On Saturdays, the prompts were sent out at 11 am, 2 pm, and 5 p.m. Overall, the MES consisted of nine prompts per participant. Students received 5€ for their participation.

### Sample

We reached 393 participants, resulting in *N*_*beeps*_ = 3537. As a cut-off criterium, we excluded participants who answered less than three beeps—including participants who had very few observations would bring noise into the analysis since it would limit the true amount of within-person variability (for a similar data cleansing approach, see Sun et al., 2021). The final sample included 347 adolescents. Depending on the analysis, the number of eligible beeps ranged between *N*_*beeps*_ = 3123 (analysis without time-lagged predictors) and *N*_*beeps*_ = 2082 (analysis of time-lagged predictors: the first beep of each day was removed to avoid lagged associations that span the night or even several days). Participants were, on average, 14.42 years old (*SD* = 1.79), and 44.3% (*N* = 156) were female. Regarding their educational level, around three-quarters of the sample visited a secondary school (*N* = 270). 42.1% indicated that German is their mother tongue (*N* = 146), which aligns with the distribution in the respective school districts.

### Measures

#### Dependent Variable

##### Response

The response is represented by a dummy-coded variable (0 = non-response, 1 = response). Non-response included prompts that were not answered at all or not answered on time. The criteria for timely response were as follows: First, participants had to respond within 2 hours of the signal being sent. Second, there had to be at least 1 hour difference between the answers of two consecutive beeps; otherwise, both measures would overlap (in this case, the second beep was coded as invalid). Throughout the study, the compliance rate was 72.2% (*n*_*beeps*_ = 2256).

#### Beep Level: Study Settings

##### Study Day

The day of the study was measured with two dummy-coded variables (*second day*, *third day*), using the first day of the study as reference category.

##### Prompt Time

Two predictors were used to identify the impact of the scheduled time of the beep. *Afternoon* was a dummy-coded variable equal to 1 when a prompt occurred in the afternoon (0 = noon/evening); *evening* equals 1 when it was the last prompt of the day (0 = noon/afternoon).

#### Beep Level: Time-Lagged Predictors

##### Affective Well-Being

We assessed participants’ affective well-being with one item (Beyens et al., 2020; “How did you feel during the last hour?” 1 = very bad, 5 = excellent,; *M* = 3.73, *SD* = 1.12).

##### Flow Experience

The level of flow at the prior beep was measured with three items adapted from the Flow Short Scale (Engeser & Rheinberg, 2008; e.g., “I was so busy that I forgot everything else around me,” *M* = 3.06, *SD* = 1.06).

##### Context

To assess the context of the last prompt, we measured the spatial context of response (0 = being outdoors, 1 = being at home) and social context (0 = being around others, 1 = being alone).

#### Person-Level Predictors

##### Well-Being

The overall well-being during the last two weeks was indicated on a four-point Likert scale using five items (according to the WHO Well-Being scale, Topp et al., 2015; e.g., “I have felt cheerful and in good spirits,” 1 = never, 4 = always). The index achieved a good internal consistency (α = .80; *M* = 2.30; *SD* = 0.67).

##### Academic Performance

We measured participants’ last semester grade in German and Math according to the Austrian grading system (1 = very good, 5 = poor). Both grades were summed up to a mean index of academic performance (*M* = 2.73, *SD* = 0.96).

##### Media Use

The general frequency of adolescents’ media use was measured with three items (e.g., “I use media on a very regular basis,” 1 = not at all, 4 = absolutely), forming a mean index (α = .86, *M* = 3.50, *SD* = 0.65). Moreover, we assessed participants’ deficient self-regulation with one item (Schnauber, 2017; “I would have a hard time using media less often,” *M* = 2.51, *SD* = 0.87).

##### Others

We further assessed participants’ age, gender, educational level (low vs. high), and their first language (1 = German, 0 = other language). Moreover, as a proxy for their socioeconomic background (Heppt et al., 2022), they were asked to indicate the number of books in their household on a five-point Likert scale (1 = no/very few books, 5 = enough books to fill three whole bookshelves, *M* = 3.18, *SD* = 1.42).

### Statistical Analyses

We used multilevel binary logistic regression models to assess the effects of the variables of interest on non-response. The multilevel statistical approach was chosen because of the nested structure of the data: The within-person variables assessed at each beep (level-1) were nested within persons (level-2).^2^ In sum, we tested five models:

- M0: random intercept only
- M1: variables related to the study settings (day, time of the prompt)
- M2: time-lagged predictors
- M3: person-level variables
- M4: overall model

We used the lme4 package (Bates et al., 2023) in the statistical software R to conduct the analyses. According to recent literature on multilevel logistic regression (Yaremych et al., 2021), we used the following centering approach: Level-1 variables were included both between-person centered and within-person centered (except for the study setting variables, which were only centered within-person since they systematically varied between persons). For all level-2 variables, we used grand-mean centering. The R script can be found on OSF (https://osf.io/mnf34/?view_only=a57d73dc3b5d4c7988872ccd9f2ded72).

## Results

The findings of the multilevel logistic regression analyses are displayed in Table 1.

**Table 1.** Predictors of Response (Multilevel Binary Logistic Regression).

	Model 1		Model 2		Model 3		Model 4	
	b	OR (95% CI)	b	OR (95% CI)	b	OR (95% CI)	b	OR (95% CI)
Intercept	1.21***	—	1.28***		1.18***		1.44***	
L1: Settings								
Day 2 (wpc)	−0.12	0.89 (0.72, 1.10)					0.08	1.08 (0.80, 1.47)
Day 3 (wpc)	−0.18	0.83 (0.68, 1.03)					0.02	1.02 (0.73, 1.45)
Afternoon (wpc)	−0.52***	0.60 (0.48, 0.73)					−0.74***	0.48 (0.37, 0.62)
Evening (wpc)^a^	0.04	1.05 (0.84, 1.30)					—	—
L1: Time-lags								
Mood (wpc)			−0.16^t^	0.84 (0.71, 1.02)			−0.18^t^	0.84 (0.69, 1.01)
Mood (bpc)			−0.00	0.10 (0.81, 1.23)			−0.02	0.99 (0.78, 1.25)
Flow (wpc)			−0.06	0.94 (0.79, 1.12)			−0.11	0.90 (0.76, 1.07)
Flow (bpc)			−0.03	0.97 (0.77, 1.23)			−0.06	0.94 (0.74, 1.19)
Alone (wpc)			−0.21	0.81 (0.57, 1.15)			−0.21	0.81 (0.57, 1.16)
Alone (bpc)			−0.06	0.94 (0.50, 1.75)			−0.14	0.87 (0.46, 1.63)
At home (wpc)			0.12	1.12 (0.83, 1.52)			−0.00	0.99 (0.71, 1.42)
At home (bpc)			0.73*	2.08 (1.02, 4.26)			0.74*	2.09 (1.00, 4.39)
L2: Ind. factors								
Age					0.01	1.01 (0.92, 1.10)	0.01	1.01 (0.91, 1.14)
Gender					−0.23	0.79 (0.59, 1.07)	−0.23	0.79 (0.55, 1.14)
Education					−0.11	0.89 (0.59, 1.36)	−0.18	0.84 (0.50, 1.39)
Native speaker					0.32	1.37 (0.98, 1.92)	0.38^t^	1.47 (0.97, 2.22)
Books-at-home					0.12*	1.13 (1.00, 1.27)	0.13^t^	1.13 (0.98, 1.32)
School grade					−0.23**	0.79 (0.68, 0.93)	−0.26**	0.77 (0.64, 0.93)
Self-regulation					−0.07	0.93 (0.78, 1.11)	−0.10	0.90 (0.73, 1.12)
Media use					−0.09	0.91 (0.71, 1.17)	−0.00	1.00 (0.73, 1.35)
Well-being					−0.10	0.91 (0.73, 1.14)	0.08	1.09 (0.80, 1.49)

*Note. N*_*Model 1*_ = 3123, *N*_*Model 2*_ = 1724, *N*_*Model 3*_ = 3078, *N*_*Model 4*_ = 1696; L1, Level-1 variables; L2, Level-2 variables; wpc, within-person centered, bpc, between-person centered.

^t^ *p <* .10, *p < .05, **p < .01, ***p < .001.

^a^since the overall model is based on the lagged data set—therefore only containing the second and third beep of the day—the *evening*-variable is displayed in the *afternoon*-dummy in M4.

### Null Model: Intercept-Only Random Effects Model

The Null Model confirms the importance of using a nested regression model, with a between-person variance of 1.13 points (*SD* = 1.06), AIC = 3493.87. The estimated effect of the random intercept is *b* = 1.19 (*SE* = 0.08), *p* < .001.

### Model 1: Study Settings

M1 analyzed the influence of day (RQ1) and scheduled time of the prompt (RQ2), AIC = 3464.93. We found no significant effect of study day on non-response (*ns*). Regarding the scheduled time of the prompt, the analysis showed a significant effect of the afternoon-dummy (χ^2^ = 23.65; df = 1; OR = 0.60; *p* < .001) but not the evening dummy (*ns*). Hence, the odds for response were significantly lower when the beep was received in the afternoon compared to noon/evening.

### Model 2: Time-Lagged Predictors

The time-lagged predictor model showed a better quality compared to model 1, AIC = 1847.10. Of all included variables, only the between-person centered spatial context had a significant impact on the compliance (χ^2^ = 4.57; df = 1; OR = 2.20; *p* < .05). Thus, adolescents who were at home at every prior beep had higher response odds than participants who were outside their home every time they received a signal. Since we found only a between-person but no within-person effect, H1a was partly supported. In contrast, the social context had no influence, therefore rejecting H1b. No impact occurred for the time-lagged affective well-being (RQ3) and flow experience (RQ4).

### Model 3: Person-Level Predictors

M3 contains the person-level variables, AIC = 3450.70. In detail, we were interested in the effects of sociodemographic (RQ5a) and socioeconomic factors (RQ5b), media use (RQ5c), academic performance (RQ5d), and overall well-being (RQ5e). Of all observed variables, only the academic performance and the socioeconomic proxy variable, books-at-home, became significant. Therefore, adolescents with poorer school grades (χ^2^ = 8.55; df = 1; OR = 0.79; *p* < .01) had lower odds of response, while those with higher socioeconomic status were more likely to respond (χ^2^ = 3.86; df = 1; OR = 1.13; *p* < .05). No effects occurred for age, gender, educational status, cultural background, media use, self-regulation, and overall well-being.

### Model 4: Overall Model

**Figure 1.**
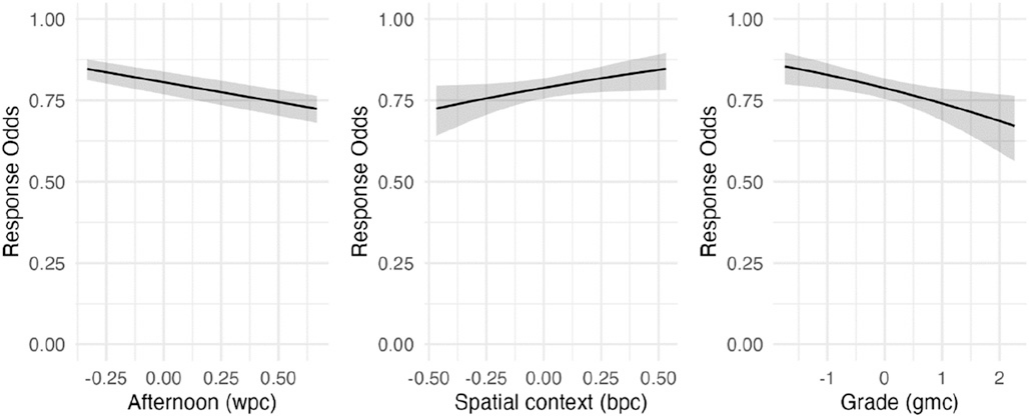
Effects of significant predictor variables (Model 4) on response odds (Odds Ratios, 95% CI). *Note.* wpc, within-person centered; bpc, between-person centered; gmc, grand-mean centered; the original scales are as follows: afternoon (0 = noon/evening, 1 = afternoon), spatial context (0 = outside, 1 = at home), mean grade (1 = very good, 5 = poor).

The overall model showed the best quality, AIC = 1815.07. In this model, the effects of the scheduled time of the prompt (χ^2^ = 31.46; df = 1; OR = 0.48; *p* < .001), academic performance (χ^2^ = 7.30; df = 1; OR = 0.77; *p* < .01), and spatial context (χ^2^ = 3.85; df = 1; OR = 2.10; *p* < .05) remained stable. In contrast, the impact of the socioeconomic proxy variable *books-at-home* became insignificant. The effects of the scheduled time, spatial context, and academic performance are displayed in Figure 1.

To ensure the robustness of our findings, we conducted additional analyses using ordinary logistic regression (OLR). The results of the OLR models, including the same set of variables as in the multilevel logistic regression models, are presented in the supplement (Table S1, OSF). This supplementary analysis allowed us to compare the outcomes of both modeling approaches and assess the stability of the observed effects. In summary, while both modeling approaches reveal consistent predictors of response likelihood (i.e., school grades, scheduled time of the prompt), the multilevel logistic regression captures the nuanced within-person and between-person effects more effectively. Moreover, when looking at the overall model (M4), the Akaike information criterion indicates that the multilevel logistic regression model has a better fit compared to the ordinary logistic regression model. This suggests that the multilevel approach provides a more valid and realistic representation of the data.

## Discussion

The MES method is a promising approach for studying displacement effects of digital media use on adolescents’ academic performance and well-being. However, this method is also characterized by extensive survey periods and multiple assessments per day, which can result in higher levels of missing data. Against this background, this study aimed to investigate factors related to the response odds in an MES study capturing adolescents’ media use, well-being, and learning activities. The study is novel concerning the following characteristics: (1) focusing on a youth sample in the context of media use and effects research, (2) integrating a time-lagged analytical approach, and (3) observing variables related to displacement and stimulation effects (e.g., academic performance, media use).

Among the predictor variables studied, three determinants significantly predicted the odds of response. These determinants were related to the study setting (i.e., scheduled time), context (i.e., being outside), and person (i.e., academic performance). The results provide important insights for researchers using the MES method to study media use and effects among adolescents. By understanding these factors, researchers can improve the design and implementation of their study to increase response rates and minimize missing data. This, in turn, can enhance the accuracy and reliability of findings, ultimately leading to a better understanding of media effects in youth samples.

Contrary to previous literature (e.g., McLean et al., 2017; Rintala et al., 2020), we found no effect of the study day, which may be due to the relatively short duration of our study, spanning only three days (e.g., most MES studies in psychology run about a week, Intille et al., 2016). Hence, the chosen duration might be a good fit when conducting MES studies among adolescents to avoid panel mortality. Moreover, the incentive of receiving €5 for participating on all study days likely motivated students to remain engaged, emphasizing the importance of maintaining motivation when conducting MES research.

Moreover, we found a U-shaped effect of prompt time, with the second prompt of the day having significantly lower response odds than those scheduled at other times (for a similar result, see Broda, 2017). This finding might be because the second prompt of the day falls exactly in the free disposable time of the observed target group. Therefore, the participants might have been distracted or busy doing other things when they received the second daily signal. The significant effect of the scheduled prompt time suggests that researchers should carefully consider the pros and cons of afternoon time slots when planning the prompt schedule. However, it can be assumed that the occurring missingness falls into the category MAR, therefore not leading to biased findings.

Our study employed a time-lagged perspective to investigate the experiences of participants when missing a beep. The good news is that—despite having a robust sample size that allowed us to test for small effect sizes—we found no significant impact of affective well-being, flow experience, and social context. The only factor that affected response odds was spatial context, which aligns with previous studies (Rintala et al., 2020; Sokolovsky et al., 2014). Specifically, adolescents who have been outside their homes during previous beeps were less likely to respond than those who were at home. However, it should be noted that we only found a between-person effect and no within-person effect. Based on our findings, it is unlikely that the relationship points towards MNAR data.

Regarding the impact of observed person-level variables, we found no influence of sociodemographic factors and overall well-being on response odds. However, we did find that mean school grade was negatively associated with the odds of a response. Given that digital media use has been linked to both stimulation and displacement effects on adolescents’ academic performance (e.g., Lacka et al., 2021; Tak & Catsambis, 2023; Zachos et al., 2018), this finding is particularly noteworthy as it indicates that the data is missing in a nonrandom manner (i.e., low-performing students have lower odds to respond). We draw two conclusions from this finding: First, researchers in this field could consider to oversample participants with lower educational status in order to compensate for the lower response odds. Second, when aiming to analyze displacement effects of digital media use on adolescents’ academic performance based on MES data, it seems highly recommendable to apply multiple imputation before analyzing the data, since this approach considers observed causes or correlates of missingness (Graham, 2009; Stone et al., 2023; Sun et al., 2021). Interestingly, to the best of our knowledge, none of the MES studies in media use and effects research have reported on how they dealt with missing data in their analyses. Consequently, it is likely that authors have been relying on complete case analysis, since this method is easy to implement and the default-option in many statistical packages (e.g., SPSS). Based on our findings, we urge researchers to carefully check if their data is actually MCAR/MAR before relying on list-wise deletion instead of applying modern missing data methods.

We also found that the socioeconomic proxy variable *books-at-home* positively predicted adolescents’ response behavior, although this effect did not reach significance in the overall model, which may be due to the limited sample size of M4 compared to M3. Still, we encourage researchers—especially when examining education-related outcomes—to measure this one-item predictor to include it as correlate of missingness when conducting multiple imputation.

Our study has some limitations that should be considered when interpreting the findings. First, our results can only provide statements about the associations between the observed Level-1 and Level-2 variables and the response odds. Other factors may also explain compliance in MES studies, such as personality traits (e.g., being disciplined or organized). Second, lagged predictor models were used, which may not be ideal as they assume that the feelings and contextual factors of the prior beep are also valid for the current prompt. To gain more detailed insights into participants’ activities, more advanced methods such as psychophysiological measures (e.g., wearables; Martinez et al., 2021) or audio-/videotaping (e.g., Sun et al., 2021) may be employed. However, these methods are time- and cost-consuming and may lead to socially desirable response patterns due to participants feeling observed. Third, the use of lagged predictor models resulted in the elimination of 33% of our sample (three of nine prompts), limiting the power of our study. Nonetheless, we believe that the time-lagged analysis is based on a substantial number of observations and that the findings remain generalizable. Fourth, our analytical strategy focused on traditional statistical methods, that is, multilevel logistic regression analysis. Based on current literature, future research could explore the potential of machine learning models for a more comprehensive understanding of the predictive factors in adolescents’ response behavior (for a similar approach, see Lopez-Larrosa et al., 2023). Lastly, while our study investigated the predictors of non-response, future studies are needed that implement a sensitivity analysis to demonstrate how the handling of MNAR data affects the findings (e.g., by comparing the results of a complete case analysis vs. multiple imputation).

## Conclusion

Our study sheds light on predictors of non-response in an MES study on displacement effects of digital media use on adolescents’ well-being and academic performance. The findings reveal that the data is not per se missing at random and strongly associated with poorer school grades. Thus, to ensure accurate findings, sophisticated missing data methods such as multiple imputation should be applied before analyzing the data. Additionally, the prompt’s scheduled time, spatial context, and socioeconomic status of the participant can also impact response rates and should be taken into account. Overall, our study highlights the significance of careful consideration of potential factors that may impact response rates to ensure valid and reliable findings in research on displacement effects among youth.

## Supplemental Material

Supplemental Material - Uncovering the Missing Pieces: Predictors of Non-Response in a Mobile Experience Sampling Study on Media Effects Among Youth Supplemental Material for Uncovering the Missing Pieces: Predictors of Non-Response in a Mobile Experience Sampling Study on Media Effects Among Youth by Anne Reinhardt, Sophie Mayen, and Claudia Wilhelm in Social Science Computer Review.

Supplemental Material - Uncovering the Missing Pieces: Predictors of Non-Response in a Mobile Experience Sampling Study on Media Effects Among Youth Supplemental Material for Uncovering the Missing Pieces: Predictors of Non-Response in a Mobile Experience Sampling Study on Media Effects Among Youth by Anne Reinhardt, Sophie Mayen, and Claudia Wilhelm in Social Science Computer Review.

## Data Availability

The data, R scripts, and supplementary material are openly accessible via OSF (https://osf.io/mnf34/?view_only=a57d73dc3b5d4c7988872ccd9f2ded72).

## Notes

1. This article is part of a research project aiming at identifying displacement effects of digital media use on adolescents’ well-being and academic performance. Notably, the research questions and hypotheses of this paper are not part of the pre-registration. However, the pre-registered sampling procedures, measures, and outlier handling also apply here.

2. We also considered integrating the study day as a second random intercept into the model; however, the model resulted in being almost singular, meaning that nesting beeps within days within participants did not result in meaningful findings.
