# Supplementary material for: Uncovering the Missing Pieces: Predictors of Nonresponse in a Mobile Experience Sampling Study on Media Effects Among Youth
Source: Soc Sci Comput Rev. 2024 Feb 23;42(6):1464–78. doi: 10.1177/08944393241235182 (PMC11549009; doi:10.1177/08944393241235182)
Supplement: Supplemental Material - Uncovering the Missing Pieces: Predictors of Non-Response in a Mobile Experience Sampling Study on Media Effects Among Youth [file sj-pdf-1-ssc-10.1177_08944393241235182.pdf]

Supplement

Table S1

Predictors of non-response (ordinary logistic regression)

	Model 1		Model 2		Model 3		Model 4	
	b	OR (95% CI)	b	OR (95% CI)	b	OR (95% CI)	b	OR (95% CI)
Intercept	1.18***	-	1.28***		0.71***		1.21***	
Day 2 (dummy)	-0.09	0.91 (0.75, 1.10)					0.07	1.07 (0.82, 1.42)
Day 3 (dummy)	-0.15	0.86 (0.71, 1.04)					-0.03	0.97 (0.71, 1.31)
Afternoon (dummy)	-0.42***	0.65 (0.54, 0.79)					-0.67***	0.51 (0.40, 0.64)
Evening (dummy) [a]	0.04	1.03 (0.85, 1.27)					-	-
Mood (gmc)			-0.06	0.94 (0.85, 1.04)			-0.09	0.91 (0.81, 1.02)
Flow (gmc)			-0.04	0.96 (0.86, 1.07)			-0.07	0.94 (0.84, 1.05)
Alone (dummy)			-0.10	0.90 (0.70, 1.16)			-0.13	0.88 (0.68, 1.13)
At home (dummy)			0.22[t]	1.25 (0.99, 1.58)			0.16	1.17 (0.89, 1.53)
Age (gmc)					0.01	1.00 (0.96, 1.06)	-0.01	0.99 (0.92, 1.07)
Gender (dummy)					-0.19*	0.82 (0.70, 0.97)	-0.18	0.84 (0.66, 1.06)
Edu (dummy)					-0.08	0.92 (0.73, 1.17)	-0.13	0.88 (0.63, 1.22)
Native sp. (dummy)					0.25**	1.29 (1.07, 1.56)	0.29*	1.34 (1.02, 1.75)
Books (gmc)					0.10**	1.11 (1.03, 1.18)	0.11*	1.13 (1.02, 1.24)
Grade (gmc)					-0.19***	0.83 (0.76, 0.90)	-0.19**	0.82 (0.73, 0.93)
Self-reg. (gmc)					-0.06	0.95 (0.86, 1.04)	-0.10	0.91 (0.79, 0.93)
Media use (gmc)					-0.08	0.92 (0.80, 1.06)	0.05	1.05 (0.86, 1.28)
Well-being (gmc)					-0.07	0.93 (0.82, 1.06)	0.10	1.10 (0.91, 1.22)
AIC	3668.9		1931.4		3623.6		1871.8	
N (observations)	3123		1724		3078		1680	

Note 1. [t] $p < .10$, * $p < .05$, ** $p < .01$, *** $p < .001$. [a] since the overall model is based on the lagged data set—therefore only containing the second and third beep of the day—the *evening*-variable is displayed in the *afternoon*-dummy in M4;

Note 2. Descriptive overview of the similarities and differences in the findings between the multilevel logistic regression (ML) and the ordinary logistic regression (OLR) for the overall model (M4): In both ML and OLR, the scheduled time of the prompt (OR = -0.74, p < .001 in ML; OR = -0.67, p < .001 in OLR) and school grade (OR = -0.23, p < .01 in ML; OR = -0.19, p < .01 in OLR) consistently emerged as significant predictors of response likelihood. Socioeconomic status (books-at-home) became tendentially significant in the multilevel model but reached significance in the ordinary logistic regression (OR = 0.13, p < .10 in ML; OR = 0.11, p < .05 in OLR). The same applied for being a native German speaker (OR = 0.38, p < .10 in ML; OR = 0.29, p < .05 in

OLR). In contrast, while spatial context was a significant predictor on the between-person level in the ML model, the uncentered predictor was insignificant in the OLR model (OR = 0.74, $p < .05$ in ML; OR = 0.16, ns in OLR). The Akaike information criterion (AIC) indicates that the multilevel logistic regression model (AIC = 1815.07) has a better fit compared to the ordinary logistic regression model (AIC = 1871.8). This suggests that the multilevel approach provides a more effective representation of the data. In summary, while both modeling approaches reveal consistent predictors of response likelihood, the multilevel logistic regression captures the nuanced within-person and between-person effects more effectively. The differences in significance levels for certain predictors, such as spatial context and socioeconomic status, underscore the importance of considering individual variations and nested data structures in our study.
